# Supplementary material for: How Do the Psychological Functions of Eating Disorder Behaviours Compare with Self-Harm? A Systematic Qualitative Evidence Synthesis
Source: Healthcare (Basel). 2025 Aug 5;13(15):1914. doi: 10.3390/healthcare13151914 (PMC12346682; doi:10.3390/healthcare13151914)
Supplement: Supplementary file 1 [file healthcare-13-01914-s001.zip › Supplementary Material - Included Papers Record.pdf]

Supplementary material S1: Included studies

Author/Year/Title	Country	Population: Number, age range and gender.	Eating disorder or behaviours investigated	Research approach and Methods	Primary aim of the study
**1.** Ålgars, M., Alanko, K., Santtila, P. and Sandnabba, N.K. 2012. Disordered eating and gender identity disorder: a qualitative study. *Eating disorders.* **20**(4), pp.300-311.	Finland	20 participants (11 female-to-male, 9 male-to-female) transgender Finnish adults, aged 21–62 years.	Dieting, Bingeing, Purging and Excessive exercise.	Semi-structured interviews and ground theory coding.	The aim of the present study was to examine eating behaviours and cognitions in a sample of transgender Finnish adults. The incidence and nature of disordered eating was investigated, as well as the participants' own understanding of its underlying causes. The possible effect of gender reassignment treatment on the level and nature of disordered eating was also investigated.
**2**. Amirapu, A. and Brady-Van den Bos, M. 2023. Disordered eating in female Indian students during the Covid-19 pandemic: The potential role of family. *International Journal of Eating Disorders.* **56**(1), pp.143-150.	UK, India, USA and Singapore	10 female undergraduate students between the ages of 10-23.	Disordered eating unspecified (quotations refer to restrictive eating and over exercise)	Semi-structured interviews and reflexive inductive thematic analysis.	To gain an understanding of how female Indian university students, who moved out of their parental home for their studies and moved back with them (either in India, UK, USA or Singapore) during lockdown experienced food and exercise disorders, with a focus on the influences of their parents and culture.
**3.** Arkell, J. and Robinson, P. 2008. A pilot case series using qualitative and quantitative methods: biological, psychological and social outcome in severe and enduring eating disorder (anorexia nervosa). *International Journal of Eating Disorders.* **41**(7), pp.650-656.	London, UK.	11 participant. The mean age was 37.7 years (SD 8 years). 10 female and 1 male.	Participants must have continuously fulfilled the criteria for an ICD-10 diagnosis of Anorexia Nervosa apart from temporary weight restoration due to hospital admission.	Mixed methods. Qualitative component = 1 hour interview and written task analysed using a thematic framework approach.	To assess in detail the level of disability and quality of life in participants who have been consistently ill with anorexia nervosa for over 10years.
**4.** Batchelder, E., Della Sega, C. and Levine, M.P. 2023. Eating Disorder blogs during Covid-19: A window into recovery progress. *Qualitative Methods in Psychology Bulletin.* **36**, pp.28-43.	USA	Multiple blogs from 9 different individuals. Due to online anonymity information about age range and gender were not documented.	Bloggers self-identified as currently having or having had an eating disorder.	Thematic analysis of online eating disorder recovery focused blogs.	To describe the response to Covid-19 of people with eating disorders via online eating disorder recovery focused blogs.
**5.** Björk, T., Wallin, K. and Pettersen, G. 2012. Male Experiences of Life After Recovery from an Eating Disorder. *Eating Disorders.* **20**(5), pp.460-468	Norway and Sweden	15 male participants aged between 19-52 years old.	10 participants had a diagnosis of anorexia nervosa, 4 bulimia nervosa and 1 had an unspecified eating disorder.	Qualitative phenomenographic approach to guide interviews and analysis.	To describe how former male patients perceive life after recovery from an eating disorder.

**6.** Blackburn, B., O'Connor, J. and Parsons, H. 2021. Becoming needless: A psychoanalytically informed qualitative study exploring the interpersonal and intrapsychic experiences of longstanding anorexia nervosa. *International Journal of Applied Psychoanalytic Studies.* **18**(4), pp.428-442.	Ireland.	6 women aged 22-44.	Anorexia Nervosa.	Psychoanalytically informed interviews and research design.	To explore the interpersonal and intrapsychic experiences of individuals with longstanding anorexia and consider how such experiences might influence barriers to recovery.
**7.** Bradley, M. and Simpson, S. 2014. Inside the experience of recovering from anorexia nervosa: An interpretative phenomenological analysis of blogs. *Counselling, Psychotherapy and Health.* **9**(1), pp.1-34.	Australia.	5 blogs. The five women who wrote the blogs were between 27 and 45 years of age, with a mean of 35 years of age.	Anorexia Nervosa.	Publicly accessible blogs via the internet analysed using interpretative Phenomenological Analysis.	This study aimed to investigate and interpret the treatment and recovery experiences in AN from the perspectives of recovering individuals.
**8.** Brede, J., Babb, C., Jones, C., Mair, E., Zanker, C., Tchanturia, K., Serpell, L., Fox, J. and Will, M. 2020. "For Me, the Anorexia is Just a Symptom, and the Cause is the Autism": Investigating Restrictive Eating Disorders in Autistic Women. *Journal of Autism and Developmental Disorders.* **50**(12), pp.4280-4296.	UK.	15 women with a diagnosis of autism and experience of anorexia nervosa, 13 parents and 16 healthcare professionals. Age ranges between 23-58.	Anorexia Nervosa.	Semi-structured interviews and thematic analysis.	To better understand how anorexia nervosa develops and persists in autistic individuals from the perspective of autistic women, parents and healthcare professionals and to derive a theoretical model of restrictive eating difficulties in autism.
**9.** Breland, J.Y., Donalson, R., Dinh, J.V. and Maguen, S. 2018. Trauma exposure and disordered eating: A qualitative study. *Women Health.* **58**(2), pp.160-174.	USA	20 women (one identified as a transgender woman). Mean age was 48 years (SD=15).	Additional screening questions ensured that all participants engaged in disordered eating	Five focus groups and two dyadic interviews. Thematic analysis.	To understand why some individuals exposed to trauma report disordered eating.
**10.** Bremer, M.F., Garnweidner-Holme, L., Nesse, L. and Molin, M. 2023. Experiences of living with binge eating disorder and facilitators of recovery processes: a qualitative study. *Journal of eating disorders.* **11**(1), p201.	Norway.	6 women between the ages of 30 and 70 years.	Binge eating disorder.	Semi-structured interviews. Analysis guided by Malterud's systematic text condensation inspired by phenomenology.	To explore how patients experience living with BED and to investigate factors perceived as facilitating recovery.
**11.** Brooks, A., LeCouteur, A. and Hepworth, J. 1998. Accounts of experiences of bulimia: a discourse analytic study. *The International journal of eating disorders.* **24**(2), pp.193-205.	Australia	10 female and one male participant between the ages of 19 and 53 years.	Bulimic behaviour.	Semi-structured interviews. Discourse analysis.	To identify the variety of versions of bulimia constructed by participants, to suggest functions and consequences of these constructions and to examine the sociocultural ideologies evident in participant discourses.

**12.** Broomfield, C., Rhodes, P. and Touyz, S. 2021. How and why does the disease progress? A qualitative investigation of the transition into long-standing anorexia nervosa. *Journal of eating disorders.* **9**, pp.1-10.	Australia	11 women between ages of 19 and 66 years.	Longstanding anorexia nervosa.	Narrative enquiry approach. Photovoice and interviews. Indictive thematic analysis.	To investigate the transition of anorexia nervosa from earlier to later stages.
**13.** Broussard, B. 2005. Women's experiences of bulimia nervosa. *Journal of advanced nursing.* **49**(1), pp.43-50.	USA	13 women aged between 18 and 36 years.	Self-reported bulimia Nervosa.	The principles of Heideggerian phenomenology guided the study. Data obtained through interviews, personal diaries and demographic questionnaires.	To interpret and understand bulimia nervosa as women experience it.
**14.** Brown, S., Opitz, M.C., Peebles, A.I., Sharpe, H., Duffy, F. and Newman, E. 2021. A qualitative exploration of the impact of COVID-19 on individuals with eating disorders in the UK. *Appetite.* **156**, p104977.	UK	10 participants. 9 participants identified as female and 1 as non-binary. Age range between 24 and 38 years.	Self-reported eating disorders. 6 participants mainly identified with Anorexia nervosa, 2 with eating disorders not otherwise specified and one with Bulimia Nervosa.	Semi-structured interviews. Thematic analysis underpinned by critical realism.	Explores the impact of COVID-19 and associated public health measures on adults with eating disorders within the UK.
**15.** Brownstone, L., Greene, K., Kelly, A., Maloul, K., Norling, N., Rockholm,H. and Izaguirre, M. 2022. "Are people thinking I'm a vector...because I'm fat?": Cisgender experiences of body, eating, and identity during COVID-19. *Body image.* **40**, pp.256-266.	USA.	31 participants aged between 19 and 57. 7 participants identified as male and 24 as female.	History of disordered eating both diagnosed and undiagnosed.17 participants reported a history of diagnosed eating disorder whist 14 reported undiagnosed but self-identified histories of eating disorders.	Semi-structured interviews and inductive codebook thematic analysis	To understand experiences of body and eating during the pandemic among a diverse (sub)clinical sample of individuals with self-reported disordered eating.
**16.** Brownstone, L. Kelly, D., Maloul, E., Dinneen, J., Palazzolo, L., Raque, T. and Greene, K. 2022. "It's just not comfortable to exist in a body": Transgender/gender nonbinary individuals' experiences of body and eating distress during the COVID-19 pandemic. *Psychology of Sexual Orientation and Gender Diversity.* **9**(4), pp.434-445.	USA	13 TGNB individuals. Most identified as gender non-binary whilst one participant identified as transgender woman, one as a GNB/trans man and one as a gender questioning cis man. Age range between 19 and 53.	History of disordered eating including binge eating and restricting.	Semi-structured zoom audio interview and online survey. Indictive, reflexive thematic analysis.	To take a closer look at the complexities of transgender and gender non-binary participants lived experiences during the pandemic while situating such experiences within their unique social contexts to address a gap in existing analyses of COVID019.

**17.** Brownstone, L., Mihas, P., Maman, S., Peterson, C, Bulik, M. and Bardone-Cone, M. 2021. Lived experiences of subjective binge eating: An inductive thematic analysis. *International Journal of Eating Disorders*. **54**(12), pp.2192-2205.	USA	14 participants age ranges between 19-65 years. 11 participants identified as cisgender women, 2 as cisgender men and 1 as gender non-binary.	Subjective binge eating.	Semi-structured phone interviews and Indictive, reflexive thematic analysis.	Research questions: how individuals describe the experience of subjective binge eating, how are subjective binge eating related to affect in moments before, during and after their occurrences and for those who experience objective binge eating how does the experience of subjective binge eating compare to objective.
**18.** Budd, G. 2007. Disordered Eating: Young Women's Search for Control and Connection. *Journal of child and adolescent psychiatric nursing.* **20**(2), pp.96-106.	Eastern United States.	15 women between ages of 18-25.	Self-reported disordered eating.	Grounded Theory/interviews.	To gain an understanding of disordered eating in adolescence. To explore the basic social process of disordered eating behaviour in adolescence from the perspective of a community sample of college women.
**19.** Busanich, R., McGannon, K.R. and Schinke, R.J. 2014. Comparing elite male and female distance runner's experiences of disordered eating through narrative analysis. *Psychology of Sport and Exercise.* **15**(6), pp.705-712.	USA and Canada	One 19 year old male and one 34 year old female.	The male participant identified as experiencing anorexia nervosa whilst the female participant reported engaging in disordered eating behaviours, notable extreme dieting, bingeing and purging and over exercising practices.	Narrative collective case study approach. Semi-structured narrative interviews. Structural and performative analysis.	To compare and contrast the disordered eating experiences of one male and one female athlete.
**20.** Button, E.J. and Warren, R.L. 2001. Living with anorexia nervosa: The experience of a cohort of sufferers from anorexia nerv15osa 7.5 years after initial presentation to a specialized eating disorders service. *European Eating Disorders Review.* **9**(2), pp.74-96	England	Initial cohort of 89 participant, with successful follow up of 36. 7 male and remainder female, mean age at presentation 24.1 and mean age at onset of ED 19.3.	Anorexia Nervosa.	Semi-structured interviews and questionnaires.	To further understand how sufferers from anorexia nervosa view the disorder, how it affects their lives and how they experience treatment and help. Secondary aim to examine the clinical, social and psychological functioning of a group of adult sufferers of anorexia nervosa some years after their initial presentation at a specialized service for the treatment of eating disorders.
**21.** Carey, J.B., Saules, K.K. and Carr, M.M. 2017. A qualitative analysis of men's experiences of binge eating. *Appetite.* **116**, pp.184-195.	USA	11 male undergraduate students, age range between 19-52	overeating episodes that were consistent with DSM-5 BED criterion A1.	Grounded theory approach to analyse semi-structured interviews.	To gather detailed information about how men experience overeating and related body image concerns, to identify common themes.

**22.** Chan, Z. and Ma, J. 2002. The secrets of self-starvation. *Journal of International Women's Studies.* **3**(2), pp.23-37.	Hong Kong	1 female 24 year old participant.	Anorexia Nervosa.	Single case study with life history approach.	The primary aim of this study is to present the patient's subjective experience of her self-starvation in her immediate socio-cultural context.
**23.** Channa, S., Lavis, A., Connor, C., Palmer, C., Newman, L. and Birchwood, M. 2019. Overlaps and Disjunctures: A Cultural Case Study of a British Indian Young Woman's Experiences of Bulimia Nervosa. *Culture, Medicine and Psychiatry.* **43**(3), pp.361-386.	England	1 female participant in her early 20's.	Bulimia nervosa.	Case study from an in-depth semi-structured interview. Interpretative phenomenological analysis.	Explores the experiences of a young British Indian woman with bulimia nervosa
**24.** Churruca, K., Ussher, J.M. and Perz, J. 2017. Just Desserts? Exploring Constructions of Food in Women's Experiences of Bulimia. *Qualitative health research.* **27**(10), pp.1491-1506.	Australia	15 women aged 18-44	Clinically significant levels of bulimic behaviours assessed using the eating disorder examination questionnaire.	Telephone and face-to-face semi-structured interviews. Thematic discourse analysis informed by social constructionist theory.	To examine constructions of food in women's accounts of enacting bulimic behaviours.
**25.** Clark, M.T.R., Manuel, J., Lacey, C., Pitama, S., Cunningham, R. and Jordan, J. 2024. 'E koekoe te Tūī, e ketekete te Kākā, e kuku te Kererū, The Tūī chatters, the Kākā cackles, and the Kererū coos': Insights into explanatory factors, treatment experiences and recovery for Māori with eating disorders – A qualitative study. *Australian & New Zealand Journal of Psychiatry.* **58**(4), pp.365-372.	New Zealand	13 Moari participants with ED and 2 Whanau (support network) members, over age of 16. No further details on age or gender	Diagnosed or self-reported eating disorders: Anorexia Nervosa, bulimia and binge eating disorder.	Semi-structured interviews conducted using Kaupapa Maori research methods and thematic analysis.	To describe explanatory factors, treatment experiences and what helps recovery from eating disorders for Maori.
**26.** Colton, A. and Pistrang, N. 2004. Adolescents' experiences of inpatient treatment for anorexia nervosa. *European Eating Disorders Review.* **12**(5), pp.307-316.	UK	19 women ranging in age from 12 to 17 years, with a mean age of 15.4 years.	Participant has a primary diagnosis of anorexia and were receiving treatment from one of two inpatient, eating disorder units for adolescents in the UK.	Semi-structured interviews and interpretative phenomenological analysis.	Aimed to provide a detailed description of how adolescents on inpatient, specialist eating disorder units view their treatment.

**27.** Coman, A., Skarderud, F. and Hofmann, B.M. 2013. A disorder of a vulnerable self: Anorexia nervosa patients' understanding of disorder and self in the context of fMRI brain scanning. *Ethical Human Psychology and Psychiatry.* **15**(2), pp.120-134.	Norway	12 young women aged between 14-21 year old.	Anorexia nervosa (clinically diagnosed).	Semi-structured interviews and thematic analysis.	To explore patient perspectives regarding the aetiology of anorexia nervosa in the context of participating in brain research for AN.
**28.** Conti, J.E. 2016. "I don't think anorexia is the way out": Reconstruction of meaning in women's narratives of anorexia nervosa over 10 years. *Journal of Constructivist Psychology.* **29**(2), pp.165-183.	Australia	9 women aged 21-44 years.	Sefl-identified or clinically diagnosed anorexia Nervosa.	Interviews and critical discursive analysis	To analyse and develop insights into some of the ways in which a group of women who identified their experiences as anorexia nervosa negotiated and renegotiated, constructed and reconstructed their identifies through dialogue over 10 years.
**29.** Cummings, M.P., Alexander, R.K. and Boswell, R.G. 2023. "Ordinary days would be extraordinary": The lived experiences of severe and enduring anorexia nervosa. *The International journal of eating disorders.* **56**(12), pp.2273-2282.	USA	17 cisgender women aged between 30-61 years.	Clinically diagnosed severe and enduring anorexia nervosa	Semi-structured interviews and inductive, reflexive approach to thematic analysis.	To understand the lived experiences of individuals with severe and enduring anorexia nervosa as articulated by patients currently admitted to treatment.
**30.** Cusack, C., Iampieri, A. and Galupo, M. 2022. “I’m Still Not Sure If the Eating Disorder is a Result of Gender Dysphoria”: Trans and Nonbinary Individuals’ Descriptions of Their Eating and Body Concerns in Relation to Their Gender. *Psychology of Sexual Orientation and Gender Diversity*. **9**(4), pp.422-433.	USA	82 adult participants (M = 24.65 years, SD = 5.32). Transgender women were the most predominant identity within this sample (29.27%), followed by nonbinary individuals (26.83%) and transgender men (19.51%).	Self-reported eating disorder, disordered eating, and/or body image concerns.	Mixed method survey. Open-ended question: “How does your eating disorder, disordered eating, or eating and/or body image concerns relate to your gender identity and expression?” Participants were able to write in a response without restrictions on length of response or time responding. Written responses comprised the data used for thematic analysis	Aimed to centre on TNB individuals in order to describe how they conceptualize their EDs, disordered eating, and/ or body image concerns in relation to their gender identity and gender expression.
**31.** Cusack, C.E., Levenson, N.H. and Galupo, M.P. 2022. “Anorexia Wants to Kill Me, Dysphoria Wants Me to Live”: Centering Transgender and Nonbinary Experiences in Eating Disorder Treatment. *Journal of LGBTQ Issues in Counseling.* **16**(3), pp.265-284.	USA and Canada	46 Trans and non-binary individuals aged between 18-41 years.	Have received treatment for an eating disorder.	Open ended questionnaires and thematic analysis	Understanding what works well, and what is harmful, for TNB clients receiving ED treatment with the goal of informing the quality of care.

**32.** D'Abundo, M. and Chally, P. 2004. Struggling with recovery: participant perspectives on battling an eating disorder. *Qualitative Health Research.* **14**(8), pp.1094-1106.	USA	20 female participants ranging in age from 17 to 46 years.	Women who had recovered from or were in the process of recovering from a self-reported eating disorder. Many participants described symptoms of both anorexia and bulimia.	In depth semi-structured interviews, participant observations (of a weekly support group) and a focus group (with 5 women 2 of which had previously been interviewed). Grounded theory and constant comparative analysis.	To explore the process of recovery in women and girls with eating disorders. The research question was: What are the research participants' perspectives about recovery from eating disorders?
**33.** Dawson, L., Rhodes, P. and Touyz, S. 2014. 'Doing the Impossible': The Process of Recovery From Chronic Anorexia Nervosa. *Qualitative Health Research.* **24**(4), pp.494-505.	Australia	8 women age range between 31 and 64 years.	Clinically diagnosed anorexia nervosa.	Narrative enquiry to collect and analyse personal narratives. In depth face-to-face interviews and paradigmatic analysis.	To explore the process of recovery over time from the perspective of those who had fully recovered from chronic anorexia nervosa using stringent recovery criteria.
**34.** Dignon, A., Beardsmore, A., Spain, S. and Kuan, A. 2006. 'Why I won't eat': patient testimony from 15 anorexics concerning the causes of their disorder. *Journal of health psychology.* **11**(6), pp.942-956.	UK	15 participants, 2 men and 13 women. No details on age range.	Clinically diagnosed anorexia.	Open ended unstructured interview. Data were collected and analysed using grounded theory.	To collect detailed patient narratives to conduct an appraisal of the reasons patients give for their illness. To enable patients to describe their disorder in their own words.
**35.** Duffy, M.E., Henkel, K.E. and Earnshaw, V.A. 2016. Transgender Clients' Experiences of Eating Disorder Treatment. *Journal of LGBT Issues in Counselling.* **10**(3), pp.136-149.	USA, Europe and Canada	84 transgender and gender-diverse participants. 7% identified as women, 36% as men and 57% non-binary. Age ranges between 18-33 years.	Self-reported eating disorders, including anorexia, bulimia, binge eating and not otherwise specified ED.	Online questionnaire and inductive thematic analysis.	To explore the extent to which current ED treatment practices meet the needs of transgender individuals, as well as what improvements could be made.
**36.** Drummond, M. 1999. Life as a Male 'Anorexic'. *Australian Journal of Primary Health.* **5**(2), pp.80-89.	Australia	8 male participants. No information given on age range.	"Eating disordered men." The paper discusses bulimia and anorexia but does not specify if participants had received diagnoses.	In depth phenomenological interviews and inductive approach to theorisation.	To provide insight into the lives of male anorexia and bulimia nervosa sufferers.
**37.** Eikey, E.V. 2021. Effects of diet and fitness apps on eating disorder behaviours: Qualitative study. *British Journal of Psychiatry.* **7**(5), pe176.	USA.	24 participants aged 18-23 years. No information on gender.	Self-identified eating disorder behaviours	Semi-structured interviews and thematic analysis.	To investigate the unintended negative consequences of engaging with diet and fitness apps amongst university and college students.

**38.** Eiring, K., Wiig Hage, T. and Reas, D.L. 2021. Exploring the experience of being viewed as "not sick enough": a qualitative study of women recovered from anorexia nervosa or atypical anorexia nervosa. *Journal of eating disorders.* **9**(1), p142.	Norway	7 females ages between 21-47 years.	Clinically diagnosed anorexia or atypical anorexia	Semi-structured interviews and interpretative phenomenological analysis.	To investigate the perspectives of individuals who have previously battled a restrictive eating disorder who were considered 'not sick enough' by others at some point during their illness, and to understand the perceived impact on the illness and recovery.
**39.** Eivors, A., Button, E., Warner, S. and Turner, K. 2003. Understanding the experience of drop-out from treatment for anorexia nervosa. *European Eating Disorders Review.* **11**(2), pp.90-107.	UK	8 women between ages 21-43.	DSM-IV criteria for anorexia nervosa.	This research draws on a range of perspectives, including social constructionism, feminism, systemic and personal construct theory. Semi-structured interviews and narrative written accounts.	To develop an understanding of the meaning of drop-out from services for individuals diagnosed with anorexia nervosa.
**40.** Eli, K. 2015. Binge eating as a meaningful experience in bulimia nervosa and anorexia nervosa: a qualitative analysis. *Journal of mental health.* **24**(6), pp.363-368.	Israel	16 women aged 18-38 years.	Clinically diagnosed anorexia or bulimia with bingeing and purging symptoms or ED not otherwise specified.	In person semi-structured Interviews and phenomenologically informed thematic analysis.	To examine how women with anorexia nervosa and bulimia nervosa interpret their experiences of binge eating.
**41.** Fox, A., Larkin, M. and Leung, N. 2011. The personal meaning of eating disorder symptoms: an interpretative phenomenological analysis. *Journal of health psychology.* **16**(1), pp.116-125.	UK	8 women aged between 18 and 30.	Self-reported experiences of eating disorders.	Semi-structured interviews and interpretative thematic analysis.	To explore the personal meaning of eating difficulties.
**42.** Fox, J. 2009. A qualitative exploration of the perception of emotions in anorexia nervosa: a basic emotion and developmental perspective. *Clinical psychology & psychotherapy.* **16**(4), pp.276-302.	UK	11 female participants age range between 18-51 years.	Clinically diagnosed anorexia nervosa.	Grounded theory methodology to gather and analyse interview data.	To examine account of individuals with a current diagnosis of anorexia focusing on their experiences and management from a basic emotions and developmental perspective.
**43.** Fox, N., Ward, K. and O'Rourke, A. 2005. Pro-anorexia, weight-loss drugs and the internet: An 'anti-recovery' explanatory model of anorexia. *Sociology of Health and Illness.* **27**(7), pp.944-971.	UK, USA, New Zealand and Australia	Mostly female between the ages of 14-42. No specific number of participants – multiple users and threads posted.	Anorexia and radical use of weight loss pharmaceuticals.	Ethnography (virtual participant observations) and in depth online interviews. Thematic analysis approaches.	To explore the use of an internet website and discussion forum where pro-ana ideology was promoted and the use of diet drugs debated from a non-medical perspective.

**44.** Fox, J.R. and Diab, P. 2015. An exploration of the perceptions and experiences of living with chronic anorexia nervosa while an inpatient on an Eating Disorders Unit: an Interpretative Phenomenological Analysis (IPA) study. *Journal of Health Psychology.* **20**(1), pp.27-36.	UK	6 female participants. The mean age of the sample was 27 (range 19 – 50).	A diagnosis of AN with an illness duration of 6+ years	Interviews and Interpretative Phenomenological Analysis.	To explore sufferer's perceived experiences of living with and being treated within an Eating Disorders Unit (EDU) for their chronic Anorexia Nervosa.
**45.** Frayn, M., Trainor, C., Lin, M., Pitts, A., Drexler, S.A., Patarinski, A.G.G. and Juarascio, A. 2022. Patient perceptions of the relationship between food insecurity and eating disorder treatment: A qualitative exploration. *The International journal of eating disorders.* **55**(3), pp.332-342.	USA	14 participants, 1 male and 13 female. Mean age 44.5 years SD 14.3.	Clinically diagnosed binge eating disorder	Interviews and reflexive thematic analysis.	To qualitatively examine 1) mechanisms by which food insecurity impacts binge eating disorder development and maintenance, 2) effects of past and/or present food insecurity on ED treatment and 3) participant recommendations for addressing food insecurity in future BED treatment.
**46.** Freedman, G., Leichner, P., Manley, R., Sandhu, P.S. and Wang, T.C. 2006. Understanding anorexia nervosa through analysis of thematic content of letters in an adolescent sample. *European Eating Disorders Review.* **14**(5), pp.301-307.	Canada	27 adolescents, mean age of population = 16.7 and mean age of onset of disorder = 14.2. No information on gender of participants.	Clinically diagnosed anorexia nervosa or eating disorder not otherwise specified.	Participant written letters coded using a grounded theory approach to analysis.	To examine the attitudes of adolescents with anorexia nervosa towards their illness, to identify similarities and differences between adolescent and an adult population with anorexia nervosa in terms of positive and negative attitudes towards the illness, and to discuss treatment implications.
**47.** Goh, A, Davis, C. and Chew, E 2022. #EatingDisorderRecovery: a qualitative content analysis of eating disorder recovery-related posts on Instagram. *Eating and Weight Disorders.* **27**(4), pp.1535-1545.	Majority of users from USA. There were participants from UK, Australia, New-Zealand and others also.	405 Instagram posts included in final analysis from 360 unique users. Majority of participants were young adolescent females with a mean age of 19 years.	Eating disorder recovery.	Instagram posts identified from ED recovery hashtags from 17-18 July 2019. Qualitative textual data analysed through descriptive inductive thematic analysis.	To gain deeper insight into ED recovery content on Instagram by analysing the questions and images found in Instagram posts for evidence of ongoing ED's and analysis of ED recovery themes.
**48.** Gustafsson, S.A., Birgitta, E., Josefine, D., Lars, K. and and Norring, C. 2009. Perceived Expectations in Daily Life Among Adolescent Girls Suffering From an Eating Disorder: A Phenomenographic Study. *Eating Disorders.* **18**(1), pp.25-42.	Sweden	18 females between age of 15 and 19 years.	Eating disorder diagnosis according to the DSM-IV. Five participants were diagnosed with anorexia nervosa (AN), one with bulimia nervosa (BN)and the remaining participants with eating disorder, not otherwise specified (EDNOS)	Interviews analysed using phenomenographic approach.	Describe the variation of perceived expectations in daily life of adolescent girls who suffer from eating disorders.

**49.** Hallward, L. and Duncan, L.R. 2021. "Compulsive exercise is a socially acceptable prison cell": Exploring experiences with compulsive exercise across social media. *The International journal of eating disorders.* **54**(9), pp.1663-1671.	No data on geographical data of participants.	881 posts – Due to anonymity no details on age and gender of participants included.	Compulsive exercise.	Social media posts related to compulsive exercise and eating disorders from Reddit, twitter, Instagram and forums were collected for 12 months. Indictive thematic analysis.	To explore experiences around compulsive exercise and eating disorders shared on social media sites.
**50.** Hannon, J., Eunson, L. and Munro, C. 2017. The patient experience of illness, treatment, and change, during intensive community treatment for severe anorexia nervosa. *Eating disorders.* **25**(4), pp.279-296.	Scotland	5 female participants aged 23-30 years.	Clinically diagnosed anorexia	Semi-structured interviews and interpretative phenomenological analysis.	To explore experiences of intensive community treatment, illness and chance among patients with severe anorexia nervosa (sAN), particularly seeking to understand the processes involved in change and inability to change.
**51.** Hardin, P.K. 2003. Shape-shifting discourses of anorexia nervosa: reconstituting psychopathology. *Nursing inquiry.* **10**(4), pp.209-217.	Interviewees from USA. Unknown for those who posted in the online message board.	Interviewees – 11 participants. No information on age or gender.	Anorexia and self-starvation.	Data from online anorexia message boards and individual interviews. Discourse analysis.	Explore how discourses and institutional practices operate to position young women who take up the subject position of wanting to be diagnosed as anorexic.
**52.** Harrison, A., Konstantara, E., Zaremba, N., Brown, J., Allan, J., Hopkins, D., Treasure, J., Ismail, K. and Stadler, M. 2023. A cognitive behavioural model of type 1 diabetes and disordered eating (TIDE) in men derived from lived experience. *Diabetic Medicine.* **40**(Supplement 1), p160.	UK.	37 men (16 with disordered eating and 11 without). Age range for group with disordered eating = 21- 52.	Self-reported diagnosed eating behaviour such as restriction, bingeing or purging and scoring >20 on the diabetes eating problems survey.	Semi-structured interviews and thematic analysis.	To develop the first cognitive behavioural model outlining the development and maintenance of disordered eating in adult men living with Type 1 diabetes to improve on previous theoretical models of Type 1 diabetes and disordered eating and to draw comparisons to women with Type 1 diabetes and disordered eating.
**53.** Harrop, E.N., Hecht, H.K., Harner, V., Call, J. and Holloway, B.T. 2023. "How Do I Exist in This Body...That's Outside of the Norm?" Trans and Nonbinary Experiences of Conformity, Coping, and Connection in Atypical Anorexia. *International journal of environmental research and public health.* **20**(2).	USA	9 TNB adults, age range 18-36.	Atypical anorexia nervosa.	Participants were followed for one year and completed semi-structured, in depth, longitudinal qualitative interviews at baseline, 6 months and 12 months. Thematic analysis.	To explore the lived experiences of TNB people with atypical anorexia by examining how gender identity impacted experiences of ED illness and (potential) recovery.
**54.** Higbed, L. and Fox, J. 2010. Illness perceptions in anorexia nervosa: a qualitative investigation. *The British journal of clinical psychology.* **49**(3), pp.307-325.	England	13 participants, age range between 16 and 53. No information on gender.	Clinically diagnosed anorexia Nervosa	Semi-structured interviews which were analysed using constructivist grounded theory.	This study explored illness perceptions in AN using a qualitative design which was not restricted by a physical illness model but focused on personal models of AN from the perspective of those experiencing the disorder.

**55.** Holmes, S. 2016. 'Blindness to the obvious'? Treatment experiences and feminist approaches to eating disorders. *Feminism & Psychology*. **26**(4), pp.464-486.	UK	15 females age range 19-45.	Have experience of receiving treatment for anorexia, BED or bulimia	Semi-structured interviews and thematic discourse analysis.	Exploring 3 key questions: 1) What role does gender play in how women understand the aetiology of their eating problems and to what extent was this theme taken up by or explored within their treatment contexts? 2) When given a space to reflect on the feminist approaches to ED's, how do participants evaluate such perspectives in relation to their own experiences of eating/body distress, and their identities as women. 3) To what extend do the participant perceive that the feminist perspective play a role in future ED treatment and in what ways might this occur.
**56.** Howard, T., Williams, M.O., Woodward, D. and Fox, J.R.E. 2023. The relationship between shame, perfectionism and Anorexia Nervosa: A grounded theory study. *Psychology & Psychotherapy: Theory, Research & Practice.* **96**(1), pp.40-55.	England	11 female participants age range between 21 and 41.	Clinically diagnosed anorexia nervosa	Semi-structured interviews analysed using constructivist grounded theory methodology.	To explore the potential relationship between shame, perfectionism and anorexia nervosa and their impact on recovery from AN.
**57.** Jenkins, J. and Ogden, J. 2012. Becoming 'whole' again: a qualitative study of women's views of recovering from anorexia nervosa. *European Eating Disorders Review*. **20**(1), pp.e23-31.	UK	15 female participants. Age range 19-49.	Diagnosis of anorexia nervosa where the participants defined themselves as recovered or in recovery.	In depth semi-structured telephone interviews. Interpretative phenomenological analysis.	To explore the process of recovery from AN from the patient's perspective as a means to gain insights into what patients believe are the active mechanisms of change for their condition.
**58.** Jeppson, J.E., Richards, P.S., Hardman, R.K. and Granley, H.M. 2003. Binge and purge processes in bulimia nervosa: a qualitative investigation. *Eating Disorders.* **11**(2), pp.115-128.	Northern Utah, USA	8 female participants ranging in age from 20-39 years.	Bulimia Nervosa.	Semi-structured interviews. Data were analysed according to the qualitative approach suggested by Glaserand Strauss (1967) and described in detail by Lincoln and Guba (1985).	To explore the nature and functions of the binge and purge processes of those who suffer from bulimia nervosa.
**59**. Jones, R.L., Malson, H.J.P. and Sexuality. 2013. A critical exploration of lesbian perspectives on eating disorders. *Psychology and Sexuality* **4**(1), pp.62 - 74.	UK	5 women aged between 18 and 27 years.	Self-identified lesbian women with a history of 'anorexia' and/or 'bulimia'	Feminist Foucauldian framework. Semi structured interviews. Feminist Foucauldian discourse analytic approach to analysis.	To present a preliminary interview-based, qualitative exploration of how young women who self-identify as lesbian and as having had an eating disorder make sense of their experiences of developing, living with and recovering from an eating disorder

**60.** Joyce, C., Greasley, P., Weatherhead, S. and Seal, K. 2019. Beyond the Revolving Door: Long-Term Lived Experience of Eating Disorders and Specialist Service Provision. *Qualitative health research.* **29**(14), pp.2070-2083.	UK	8 participants age ranges between 20-64. No data on gender.	Self-reported eating disorders	Interviews and narrative analysis	Explores the following 1) what are peoples experiences of receiving input from services for long term ED's? 2) what are the social, political and cultural narratives which impact those experiences?
**61.** Kinnear, A., Siegel, J.A., Masson, P.C. and Bodell, L.P. 2023. Functions of disordered eating behaviors: a qualitative analysis of the lived experience and clinician perspectives. *Journal of eating disorders.* **11**(1), p141.	Canada	16 individuals who engage in DE behaviours and 14 clinicians who treat ED's. Age range of participants who engaged in DE behaviour = 18-32. 14 identified as women, 1 as a man and 1 'uncomfortably female'	Self-identified disordered eating behaviours.	Interviews and thematic analysis.	To investigate and categorise the various functions of disordered eating behaviours from the perspectives of adults who engage in DE behaviours and clinicians who treat ED's.
**62.** Kirsten, D.K. and du Plessis, W. 2008. Lived experiences of subclinical eating disorder: Female students' perceptions. *Journal of psychology in Africa.* **18**(4), pp.561-572.	South Africa	30 undergraduate females aged between 18 and 22.	Subclinical eating disorder.	Focus group interviews, drawings, letter writing and self-reflective researcher field notes. Constant comparative text analysis.	To obtain an "insider's perspective" on the lived experience of subclinical eating disorder (SED) in female university students.
**63.** Kolnes, L.-J. 2016. 'Feelings stronger than reason': conflicting experiences of exercise in women with anorexia nervosa. *Journal of eating disorders.* **4**, p6.	Norway.	6 women who were undergoing intensive in- or out- patient treatment for anorexia nervosa. Over the age of 18 (no further details available on age	Excessive exercise in Anorexia Nervosa	Interpretative phenomenological analysis (IPA). Semi-structured interviews.	To examine how patients with anorexia nervosa, understand and make sense of the experience of exercise in the context of their lives and treatment programmes.
**64**. Kolnes, L.-J. and Rodriguez-Morales, L. 2016. The meaning of compulsive exercise in women with anorexia nervosa: An interpretative phenomenological analysis. *Mental Health and Physical Activity.* **10**, pp.48-61.	Norway.	6 females, four of whom were former athletes, at different stages in their current treatment programme for anorexia nervosa in hospital units. Over the age of 18 (no further details available on age).	Compulsive exercise in Anorexia Nervosa.	Interpretative phenomenological analysis (IPA). Semi-structured interviews.	To explore the experience of compulsive exercise in women with anorexia nervosa, restrictive type, and the sense they make of these experiences in the context of their daily lives and treatment programme.

**65.** Konstantellou, A., Hale, L., Sternheim, L., Simic, M. and Eisler, I. 2019. The experience of intolerance of uncertainty for young people with a restrictive eating disorder: a pilot study. *Eating and weight disorders.* **24**(3), pp.533-540.	England.	13 young people with a restrictive eating disorder recruited from multi-family therapy groups. Aged between 12 and 18 years. 12 female participants and one male.	Anorexia Nervosa or eating disorder not otherwise specified – restrictive subtype.	Interpretative phenomenological analysis (IPA). Focus groups.	To advance our understanding of the relationship between intolerance of uncertainty and restrictive eating disorders by providing insight into young people's subjective experiences of uncertainty.
**66.** Krafchek, J. and Kronborg, L. 2019. Academic emotions experienced by academically high-achieving females who developed disordered eating. *Roeper Review.* **41**(4), pp.258-272.	Australia.	Purposive sample of 14 women, aged between 18 and 50 years, who self-identified as being academically high achieving females who had developed an eating disorder in the past but were now recovered.	Disordered eating: Participants varied in their behaviours, but the sample included experiences of restrictive eating, binge eating and purging.	Interviews. Content analysis.	To investigate the academic emotions experienced by a sample of academically high-achieving females who developed symptoms of disordered eating when they were in high school. To investigate the emotions associated with eating described by these participants to look for any similarities between the emotions that motivate academic achievement and the emotions associated with disordered eating.
**67**. Kyriacou, O., Easter, A. and Tchanturia, K. 2009. Comparing views of patients, parents, and clinicians on emotions in anorexia: a qualitative study. *Journal of Health Psychology.* **14**(7), pp.843-854.	UK	6 female participants aged between 21 and 36 years. Mean age = 26.8	The patient group consisted of current AN inpatients. Four of the patients suffered from restricting AN and two from binge/purge AN.	Focus groups and thematic analysis.	To establish what the most commonly experienced problems are relating to emotional processing in patients with AN from the perspective of patients with AN, their parents and clinicians. Specifically, we aim to explore what patients, parents, and clinicians identify as the most salient issues regarding emotions and social cognition and find possible similarities and differences in accounts across these groups
**68.** LaMarre, A., Hellner, M., Silverstein, S., Baker, J.H., Urban, B., Yourell, J., Wolfe, H., Perry, T. and Steinberg, D. 2024. "It's like building a new person": lived experience perspectives on eating disorder recovery processes. *Journal of eating disorders.* **12**(1), p96.	USA.	27 participants in total. 14 peer mentors and 13 family mentors. Participants predominantly identified as White, cisgender women; some participants identified as non-binary or as men. No information on age range.	History on an eating disorder (no further information given).	Semi-structured interviews. Reflexive thematic analysis.	To explore and generate nuanced understanding of recovery experiences of people with a lived eating disorder experience (first hand or as a caregiver) who were working as mentors in the field.

**69**. Lamoureux, M. M. H., & Bottorff, J. L. 2005. Becoming the real me: Recovering from anorexia nervosa. *Health Care for Women International*. **26**, 170–188.	British Columbia, Canada	9 women ranging from 19-48 years of age.	Anorexia. 8 reporting the restricting subtype and 1 reporting the binge-purge subtype. All were self-defined as recovered.	Grounded theory methods were used to guide data collection and analysis. In depth, open ended interviews conducted either in person or via telephone. Grounded theory analysis.	To describe the experience of recovery from anorexia nervosa from the patient's perspective in a process-oriented manner.
**70**. Latzer, Y., Edelstein-Elkayam, R., Rabin, O., Alon, S., Givon, M. and Tzischinsky, O. 2024. The Dark and Comforting Side of Night Eating: Women's Experiences of Trauma. *Psychiatry International.* **5**(1), pp.15-26.	Israel	18 women aged 19-60.	Clinically diagnosed night eating syndrome	Semi-structured interviews and thematic analysis.	To describe NES (night eating syndrome) experience, awareness, narratives and behaviour from the perspectives of patients with NES in light of their history of traumatic life events.
**71**. Lavis, A. 2018. Not eating or tasting other ways to live: A qualitative analysis of 'living through 'and desiring to maintain anorexia. *Transcultural psychiatry.* **55**(4), pp.454-474.	England	**Study 1:** 60 participants – no demographic data.<br><br>**Study 2:** 30 participants – no demographic data	Anorexia, Bulimia and EDNOS.	Participant observations and interviews. Iterative thematic analysis.	**Study 1:** Explore participants feelings towards their eating disorder.<br><br>**Study 2:** To explore risk factors for eating disorders.
**72.** Leonidas, C. and Santos, M.A. 2017. Emotional meanings assigned to eating disorders: Narratives of women with anorexia and bulimia nervosa. *Universitas Psychologica.* **16**(4), pp.1-13.	Brazil	12 women in treatment for ED, age range = 20 – 40.	Clinically diagnosed purging type AN and bulimia nervosa.	Semi-structured interview and thematic content analysis.	To investigate emotional meanings assigned to eating disorders through the narratives of women affected with these psychopathological conditions.
**73**. Lindgreen, P., Willaing, I., Clausen, L., Ismail, K., Grønbæk, H.N., Andersen, C.H., Persson, F. and Cleal, B. 2024. "I Haven't Told Anyone but You": Experiences and Biopsychosocial Support Needs of People with Type 2 Diabetes and Binge Eating. *Qualitative Health Research.* **34**(7), pp.621-634.	Denmark	20 participants age range between 31 and 76. 65% of the participants identified as female.	Self-reported disordered eating.	Semi-structured individual interviews and interpretative description analysis.	To gain in-depth insights into the experiences and biopsychosocial support needs of women and men with T2D and binge eating.

**74.** Lindstedt, K., Neander, K., Kjellin, L. and Gustafsson, S.A. 2018. A life put on hold: adolescents' experiences of having an eating disorder in relation to social contexts outside the family. *Journal of multidisciplinary healthcare*. **11**, pp.425-437.	Sweden	15 adolescents – 14 women and 1 man. Age range = 13 -18	Experience of receiving treatment for anorexia nervosa or EDNOS with predominantly restrictive symptomology	Interviews and thematic analysis.	To investigate how adolescents with experience of a restrictive eating disorder described their illness and their time in treatment in relation to social contexts outside the family.
**75.** Lord, V.M., Reiboldt, W., Gonitzke, D., Parker, E. and Peterson, C. 2018. Experiences of recovery in binge-eating disorder: a qualitative approach using online message boards. *Eating and weight disorders*. **23**(1), pp.95-105.	Unclear – forum for data collection was worldwide.	681 messages from 65 participants. Due to anonymity no demographic information.	Binge eating – may or may not have clinical diagnosis.	Data collected from a pro-recovery website. Thematic analysis	To investigate changes in thinking of BED sufferers who were able to recover from the disorder, understand more fully how guilt and self-blame affect recovery and explore the perceived motivators and challenges to recovery.
**76.** Lyons, G., McAndrew, S. and Warne, T. 2019. Disappearing in a Female World: Men's Experiences of Having an Eating Disorder (ED) and How It Impacts Their Lives. *Issues in mental health nursing*. **40**(7), pp.557-566.	UK	7 men aged 23-24 years old.	Clinically diagnosed ED or self-reported experience of ED.	Narrative interviews with narrative and thematic analysis.	To investigate the lived experiences of men diagnosed with an ED and its impact on everyday aspects of their lives.
**77.** Lyons, M.A. 1998. The phenomenon of compulsive overeating in a selected group of professional women. *Journal of advanced nursing*. **27**(6), pp.1158-1164.	USA	6 women aged between 25-55.	Self-reported binge eating behaviours.	Open ended interviews analysed suing the Giorgi (1979) method of phenomenology.	To determine the meaning of compulsive overeating (or binge eating) in the lives of adult professional women.
**78.** Ma, J. and Chan, Z. 2003. The different meanings of food in Chinese patients suffering from anorexia nervosa: implications for clinical social work practice. *Social Work in Mental Health*. **2**(1), pp.47-70.	China	34 adolescent and young women aged between 12-25.	Anorexia nervosa	Clinical observations and case vignettes. Unclear approach to analysis.	To investigate different meanings of food in families with a daughter suffering from AN in a Chinese context.
**79.** Ma, J., Chow, M., Lee, S. and Lai, K. 2002. Family meaning of self-starvation: Themes discerned in family treatment in Hong Kong. *Journal of Family Therapy*. **24**(1), pp.57-71.	China	5 AN families – 6 AN patients, all female aged 15-28.	Self-starvation in Anorexia Nervosa	Multiple case study design. Transcription of treatment sessions. Content analysis.	To identify themes from the treatment of chines families with an adolescent or an adult member suffering from anorexia nervosa through a multiple case study.

**80**. Major, L., Viljoen, D. and Nel, P. 2019. The experience of feeling fat for women with anorexia nervosa: An interpretative phenomenological analysis. *European Journal of Psychotherapy & Counselling*. **21**(1), pp.52-67.	UK	7 women, age range = 18-50	Clinically diagnosed Anorexia nervosa.	Semi-structured interviews and interpretative phenomenological analysis.	Exploring the lived experience of feeling fat for women with a diagnosis of AN.
**81**. McAulay, C., Dawson, L., Mond, J., Outhred, T. and Touyz, S. 2021. “The Food Matches the Mood”: Experiences of Eating Disorders in Bipolar Disorder. *Qualitative Health Research*. **31**(1), pp.100-112.	Australia	9 participants – Mean age 48.78 SD 15.11. 9 female and 1 male.	Self-reported ED behaviours	Interviews and thematic analysis.	To explore the complexities of the experiences of people with BD and ED’s with the aim of capturing rich and nuanced data.
**82.** McCombie, C., Ouzzane, H., Schmidt, U. and Lawrence, V. 2024. 'Physically it was fine, I'd eat what normal people do. But it's never like this in my head': A qualitative diary study of daily experiences of life in recovery from an eating disorder. *European eating disorders review: the journal of the Eating Disorders Association*. **32**(1), pp.46-55.	UK	14 participants, 2 males and 12 females. Age range unclear.	Clinically diagnosed ED	Qualitative diary app and reflexive thematic analysis.	To investigate daily experiences of living in recovery from an ED.
**83.** McNamara, C., Chur-Hansen, A. and Hay, P. 2008. Emotional responses to food in adults with an eating disorder: a qualitative exploration. *European Eating Disorders Review*. **16**(2), pp.115-123.	Queensland, Australia	10 adult females. The mean age of the group was 29.1 (18–41) years.	Five participants were diagnosed with bulimia nervosa, three were suffering from EDNOS and two from anorexia nervosa.	Semi-structured one-to-one interview and framework approach to analysis.	Examine emotional reactions to food by asking people with disordered eating to articulate their thoughts whilst viewing images of different foodstuffs.
**84.** Mincey, K.A. and Michelle Hunnicutt Hollenbaugh, K. 2022. Exploring the Experiences of People who Engage with Pro-eating Disorder Online Media: A Qualitative Inquiry. *International journal for the advancement of counselling*. **44**(4), pp.660-679.	International	8 female and 2 male participants, mean age = 23.7 (SD 4.7).	People who engage with ED online media – participants experienced body dissatisfaction, harsh eating attitudes and behaviours.	Interviews and document analysis based on phenomenology.	Explore the experiences of an international sample of adults engaging with pro-eating disorder online media.

**85.** Mirabella, M., Giovanardi, G., Fortunato, A., Senofonte, G., Lombardo, F., Lingiardi, V. and Speranza, A.M. 2020. The body i live in. Perceptions and meanings of body dissatisfaction in young transgender adults: A qualitative study. *Journal of Clinical Medicine*. **9**(11), pp.1-15.	Italy	36 participants aged between 18 and 30 years at stage T0 of hormone treatment.	Self-reported disordered eating patterns.	Clinical diagnostic interview and consensual qualitative research methodology	To explore experiences related to body dissatisfaction and investigate the issues associated with living in a body perceived as incongruent for individuals with GI (Gender incongruence).
**86.** Mitchison, D., Dawson, L., Hand, L., Mond, J. and Hay, P. 2016. Quality of life as a vulnerability and recovery factor in eating disorders: a community-based study. *BMC Psychiatry*. **16**(1), p328.	Australia	19 women age ranges between 25-53.	History of ED's, probable diagnosis determined by EDE-Q scores. Multiple behaviours present.	Semi-structured interviews and thematic analysis.	To explore individual sufferers' perspectives on QoL on the onset, maintenance and/or remission of ED symptoms.
**87.** Moola, F.J., Gairdner, S. and Amara, C. 2015. Speaking on behalf of the body and activity: Investigating the activity experiences of Canadian women living with anorexia nervosa. *Mental Health and Physical Activity*. **8**, pp.44-55.	Canada	11 Canadian women between the ages of 18 and 40.	Self-identified as living with or recovered from Anorexia Nervosa.	Phone interviews and thematic analysis	Explore how 11 Canadian women living with or recovering from AN experience activity over the course of a long illness journey
**88.** Moola, F.J. and Norman, M.E. 2017. On judgement day: Anorexic and obese women's phenomenological experience of the body, food and eating. *Feminism & Psychology*. **27**(3), pp.259-279.	Canada	19 women (9 identified as anorexic and 10 as obese) Mean age of those identifying as anorexic was 30.4 and obese = 43.9 years.	Self-identified anorexia and obesity	Semi-structured in depth interviews and analysis based on phenomenology.	Exploring how women living with anorexia and obesity in two Canadian provinces experiences the body, food and eating.
**89.** Musolino, C., Warin, M. and Gilchrist, P. 2018. Positioning relapse and recovery through a cultural lens of desire: A South Australian case study of disordered eating. *Transcultural Psychiatry*. **55**(4), pp.534-550.	Australia	28 women age ranges 19-52.	Self-identified disordered eating or diagnosed ED with delayed help seeking.	Mixed methods – Ethnographic fieldwork, focus groups, interviews and psychological evaluation. Thematic analysis.	Explores how desire operates in the daily lives of women with disordered eating.
**90.** Musolino, C., Warin, M., Wade, T. and Gilchrist, P. 2016. Developing shared understandings of recovery and care: a qualitative study of women with eating disorders who resist therapeutic care. *Journal of Eating Disorders*. **4**(1), p36.	Adelaide, South Australia	28 women. Unclear age range.	6 participants had a previous eating disorder diagnosis (anorexia nervosa) from a health care professional. The other nineteen participants had not	Mixed methods approach of ethnographic fieldwork and psychological evaluation. Semi-structured interviews, observations, field notes and the Eating Disorder	Explores the differing perspectives of recovery and care of people with disordered eating. We consider the views of those who have not sought help for their disordered eating, or who have been given a diagnosis but have not engaged with health care services. Our aim is to demonstrate the

			previously sought professional help and had never received a diagnosis.	Examination were the primary forms of data collection. Data was analysed using thematic analysis.	importance of the cultural context of care and how this might shape people's perspectives of recovery and openness to receiving professional care.
**91.** Newton, M., Boblin, S., Brown, B. and Ciliska, D. 2005. 'An engagement-distancing flux': Bringing a voice to experiences with romantic relationships for women with anorexia nervosa. *European Eating Disorders Review*. **13**(5), pp.317-329.	Canada	11 women age range 19-42.	Clinically diagnosed AN or EDNOS.	In depth semi-structure interviews. Data collection and analysis based on phenomenology.	To describe the subjective experiences of romantic relationships for women with anorexia nervosa.
**92.** Nilsson, K., Abrahamsson, E., Torbiornsson, A. and Hagglof, B. 2007. Causes of Adolescent Onset Anorexia Nervosa: Patient Perspectives. *Eating Disorders*. **15**(2), pp.125-133.	Sweden	1st admission median age 15, 1st follow up median age 23 and 2nd follow up 30. Representative clinical sample (no number given) no details on gender.	Clinically diagnosed AN.	Semi-structured interviews with AN patients 8 and 16 years after initial admission to child and adolescent clinics. Content analysis.	To describe the perspectives of former AN patients with respect to why they believed they developed AN
**93.** Nordbao, R., Espeset, E., Gulliksen, K.S., Skarderud, F., Geller, J. and Holte, A. 2012. Reluctance to recover in anorexia nervosa. *European Eating Disorders Review*. **20**(1), pp.60-67	Norway	36 women aged 18-39.	Clinically diagnosed AN.	In depth interviews based and open thematic coding. Data collection and analysis based on phenomenology and grounded theory.	To systematically explore what AN patients describe as interfering with their wish to recover
**94.** Nordbo, R., Espeset, E., Gulliksen, K.S., Skarderud, F. and Holte, A. 2006. The Meaning of Self-Starvation: Qualitative Study of Patients' Perception of Anorexia Nervosa. *International Journal of Eating Disorders*. **39**(7), pp.556-564.	Norway	18 women aged 20-34	Clinically diagnosed AN.	Interviews and phenomenological analysis	To systematically explore the meaning that patients with AN attribute to their anorectic behaviour.
**95.** O'connell, J.E., Bendall, S., Morley, E., Huang, C. and Krug, I.J.C.P. 2018. Delusion-like beliefs in anorexia nervosa: An interpretative phenomenological analysis. *Clinical Psychologist*. **22**(3), pp.317-326.	Australia	5 female participants age range 19-51.	Clinically diagnosed AN.	Semi-structured interviews and interpretative phenomenological analysis.	To qualitatively analyse the content of thoughts, beliefs and perspectives held by individuals with AN that may be of delusional intensity. It further aimed to explore the lived experiences that result from holding such beliefs.

**96.** Palmberg, A., Stern, M., Kelly, N.R., Bulik, C., Belgrave, F.Z., Trapp, S.K., Hofmeier, S.M. and Mazzeo, S.E. 2014. Adolescent Girls and Their Mothers Talk About Experiences of Binge and Loss of Control Eating. *Journal of Child and Family Studies.* **23**(8), pp.1403-1416.	USA	5 focus groups with 19 adolescent girls (mean age 14.52 SD 1.17). Separate focus groups were held with 19 mothers.	Self-reported binge and loss of control eating.	Focus groups and thematic qualitative analysis.	To examine the perceptions of White and African American adolescent girls and their mothers regarding experiences of binge and loss of control eating.
**97.** Papathomas, A. and Lavallee, D. 2012. Narrative Constructions of Anorexia and Abuse: An Athlete's Search for Meaning in Trauma. *Journal of Loss & Trauma.* **17**(4), p293.	England	6 unstructured life history interviews with one female participant aged 24.	Clinically diagnosed anorexia nervosa.	Life history interviews and narrative analysis	The study aims evolved from one centred on understanding athletes' experiences of eating disorders to one that addressed athletes' experiences of eating disorders and sexual abuse.
**98.** Patching, J. and Lawler, J. 2009. Understanding women's experiences of developing an eating disorder and recovering: A life-history approach. *Nursing Inquiry.* **16**(1), pp.10-21.	Australia	20 women age range 24-50	Anorexia nervosa and bulimia nervosa	Life history interviews and thematic analysis.	To develop an understanding of eating disorders which can be clearly differentiated from the predominantly medical perspective currently held
**99.** Pettersen, G., Rosenvinge, J. and Wynn, R. 2010. Eating disorders and psychoeducation-Patients' experiences of healing processes. *European Psychiatry.* **25**(SUPPL. 1).	Norway	13 female participants age range 18-54.	Suffering from ED (Either AN or BN) for minimum of 3 years.	Qualitative interviews and content analysis	Examine the role and impact of psychoeducative programmes and whether patients experience such programmes as helpful in their healing process.
**100.** Petersson, S., Gullbing, L. and Perseius, K.-I. 2021. Just like fireworks in my brain-A Swedish interview study on experiences of emotions in female patients with eating disorders. *Journal of Eating Disorders.* **9**.	Sweden	9 participants age range between 19-43.	Varying clinically diagnosed ED diagnoses – 2 participants with AN, one with BN and 6 with EDNOS (including 5 with atypical AN and low frequency BN).	Interviews and thematic analysis	To explore experiences of emotions by a transdiagnostic sample of patients with eating disorders.
**101.** Pettersen, G., Thune-Larsen, K.B., Wynn, R. and Rosenvinge, J.H. 2013. Eating disorders: Challenges in the later phases of the recovery process: A qualitative study of patients' experiences *Scandinavian Journal of Caring Sciences.* **27**(1), pp.92-98.	Norway	13 female participants 18-54 years.	Suffering from ED (Either AN or BN) for minimum of 3 years.	Interviews and content analysis.	To describe how patients experience the later recovery phases of eating disorders.

**102.** Petersson, S., Johnsson, P. and Perseius, K.-I. 2017. A Sisyphean task: Experiences of perfectionism in patients with eating disorders. *Journal of Eating Disorders.* **5**.	Sweden	15 female participants age range 18-44 years.	Varying clinically diagnosed ED diagnoses – 4 participants with AN, 4 with BN 7 OSFED (including 6 with atypical AN and 1 low frequency BN).	In depth semi-structured interviews and thematic analysis.	Explored descriptions and experiences of perfectionism described by a transdiagnostic sample of patients.
**103.** Proulx, K. 2008. Experiences of Women with Bulimia Nervosa in a Mindfulness-Based Eating Disorder Treatment Group. *Eating Disorders.* **16**(1), pp.52-72.	USA	6 college aged women (no specific age range given)	Clinically diagnosed bulimia nervosa	Phenomenological approach to interviews and data analysis.	Examine the experience of 6 college age women with bulimia nervosa following participation in an 8 week mindfulness based ED treatment group.
**104.** Pzetry, N., Vasconcelos, F.A.G. and Costa, L. 2017. Feelings and perceptions of women recovering from anorexia nervosa regarding their eating behavior. *Cadernos de saude publica.* **33**(9), pe00048716.	Brazil	3 female participants ages 21, 23 and 24 years.	Anorexia Nervosa.	Phenomenological approach to data collection (semi-structured interviews) and analysis.	Investigated individual perceptions and feelings of 3 women recovering from AN.
**105.** Rance, N., Clarke, V. and Moller, N. 2017. The anorexia nervosa experience: Shame, Solitude and Salvation. *Counselling & Psychotherapy Research.* **17**(2), pp.127-136.	England	12 women age range between 18-50.	AN (11 participants clinically diagnosed and 1 had a history of dietary restriction but no diagnosis as she had not sought help from the NHS).	Semi-structured interviews and thematic analysis.	To 'give voice' to the lived experience of women with AN
**106.** Redenbach, J. and Lawler, J.J.C.N. 2003. Recovery from disordered eating: What life histories reveal. *Contemporary Nurse.* **15**(1-2), pp.148-156.	Australia	5 women aged 24-51.	Anorexia and Bulimia.	Life history interviews – unclear data analysis approach	To gain a greater understanding of women's perceptions of developing, living with and recovering from an eating disorder.
**107.** Reid, M., Burr, J., Williams, S. and Hammersley, R. 2008. Eating disorders patients' views on their disorders and on an outpatient service: a qualitative study. *Journal of health psychology*. **13**(7), pp.956-960.	England	20 participants age range 17-41. One male and 19 female.	Clinically diagnosed anorexia or bulimia.	Semi-structured interviews and thematic analysis.	To determine sufferers' views of outpatient treatment for eating disorders and provide practical recommendations for treatment practice.

**108.** Reingold, O.H. and Goldner, L. 2023. "It was wrapped in a kind of normalcy": The lived experience and consequences in adulthood of survivors of female child sexual abuse. *Child abuse & neglect.* **139**, p106125.	Israel	15 participants, 12 women, 2 men and 1 transgender person. Age range = 21-50.	'Eating disorders'	Semi-structured interviews and interpretative phenomenological analysis.	To examine the lived experiences and the long-term consequences of child sexual abuse committed by women.
**109.** Rich, E. 2006. Anorexic dis(connection): managing anorexia as an illness and an identity. *Sociology of Health and Illness.* **28**(3), pp.284-305.	UK	7 young women were involved in the interviews. Age range 11-25.	Mixed eating disorder presentations.	Narratives from semi-structured interviews. Data are taken from an ethnographic project exploring the relationship between disordered eating and schooling at a leading centre in the UK. Feminist post-structuralist form of analysis.	Explores the ways in which young women 'manage' the complexities of the presentation of an anorexic identity, the stigma attached to it, and the relationships that are developed with fellow sufferers
**110.** Robinson, K.J., Mountford, V.A. and Sperlinger, D.J. 2013. Being men with eating disorders: perspectives of male eating disorder service-users. *Journal of Health Psychoogyl.* **18**(2), pp.176-186.	London, UK	8 men. Mean age was 38 (range 24–56 years).	Three participants with anorexic presentations (AN) or eating disorder not otherwise specified (EDNOS), AN subtype), five with bulimic presentations (BN or EDNOS-BN).	Semi-structured interview and interpretative phenomenological analysis.	This study aimed to address the following questions:1. What is it like for men to live with an ED? 2. What is it like for men to seek treatment for an ED? 3.What is it like for men to receive treatment for an ED?
**111.** Robinson, P. H., et al. (2015). "Severe and enduring anorexia nervosa (SEED-AN): a qualitative study of patients with 20+ years of anorexia nervosa." European Eating Disorders Review **23**(4): 318-326.	North London, UK	8 participants. 6 female and 1 male. Mean age of participants was 50 years.	A continuous history of Anorexia Nervosa, meeting Diagnostic and Statistical Manual of Mental Disorders, Fifth Edition criteria for at least the previous 20 years apart from temporary normalization of body mass index (BMI) as inpatients.	Multiple in-depth interviews and thematic analysis.	To better understand SEED (severe enduing eating disorder), we interviewed individual men and women with anorexia nervosa of long duration and undoubted severity, systematically detailing symptoms, behaviours and experiences.
**112.** Rodgers, R.F., Fischer, L.E., Laveway, K., Laws, K. and Bui, E. 2022. Fear of fatness and desire for thinness as distinct experiences: A qualitative exploration. *The International journal of eating disorders.* **55**(4), pp.530-540.	USA	29 young women age range between 18 and 25.	Disordered eating behaviours assessed using the eating disorder diagnostic scale.	Interviews and qualitative thematic analysis	To explore the subjective experiences of fear of fatness and drive for thinness in young women with body image concerns.

**113.** Roer, G.E., Solbakken, H.H., Abebe, D.S., Aaseth, J.O., Bolstad, I. and Lien, L. 2021. Inpatients experiences about the impact of traumatic stress on eating behaviours: an exploratory focus group study. *Journal of eating disorders.* **9**(1), p119.	Norway	13 female and 2 male inpatients age range 28-62 years.	Eating behaviour	Qualitative explorative focus groups. Systematic text condensation.	To explore inpatients' lived experiences of the impact of traumatic stress on eating behaviour.
**114.** Romito, M., Salk, R.H., Roberts, S.R., Thoma, B.C., Levine, M.D. and Choukas-Bradley, S. 2021. Exploring transgender adolescents' body image concerns and disordered eating: Semi-structured interviews with nine gender minority youth. *Body Image.* **37**, pp.50-62.	USA	9 participants that identified as transgender (with any gender identity that differed from their sex assigned at birth). Age ranges from 16-20 years old (mean age 17).	Disordered eating. All participants reported engaging in at least one behaviour to change their weight or shape.	Semi-structured interviews and a holistic multiple-case study design.	To explore the intersection of transgender identity, body image, and disordered eating through semi-structured interviews.
**115.** Ross, J.A. and Green, C. 2011. Inside the experience of anorexia nervosa: A narrative thematic analysis. *Counselling & Psychotherapy Research.* **11**(2), pp.112-119.	England	2 women (over age of 18 – no further information).	Clinically diagnosed AN.	Interviews and narrative thematic analysis.	Considers the question whether inpatient admission for AN was a therapeutic experience for 2 women with chronic AN
**116.** Rossotto, E., Rorty-Greenfield, M. and Yager, J. 1996. What causes and maintains bulimia nervosa? Recovered and non-recovered women's reflections on the disorder. *Eating Disorders: The Journal of Treatment & Prevention.* **4**(2), pp.115-127.	USA	80 participants (40 recovered and 40 non-recovered). All female participants aged between 18-35.	Clinically diagnosed BN	Semi-structured interviews. Coded according to a categorical system developed by the authors.	To compare recovered and non-recovered bulimic women's perceptions of the cause and maintenance of their disorder.
**117.** Santopinto, M. 1989. The relentless drive to be ever thinner: a study using the phenomenological method. *Nursing Science Quarterly.* **2**(1), pp.29-36.	Unclear	2 women between ages of 18 and 35.	ED behaviours carried out in relation to drive for thinness	Written and verbal accounts from 2 women analysed using the Giorgi modification of the phenomenological method.	Addresses the following question: what is it like to live the experience of the relentless drive to be ever thinner?
**118.** Scutt, E., Langdon-Daly, J. and Smithson, J. 2022. Experiences of eating difficulties in siblings of people with anorexia nervosa: a reflexive thematic analysis. *Journal of eating disorders.* **10**(1), p123.	UK	10 participants aged between 21 and 33 years. No information on gender.	Self-reported restrictive eating (with or without increased exercise and purging).	Semi-structured interviews and reflexive thematic analysis	To explore the experiences of siblings of people with AN who have had eating difficulties themselves and investee issues that may be important to the development and prevention of eating difficulties in this population.

**119.** Serpell, L. and Treasure, J. 2002. Bulimia nervosa: Friend or foe? The pros and cons of bulimia nervosa. *International Journal of Eating Disorders.* **32**(2), pp.164-170.	UK	54 letters from 30 female patients (mean age 19.5years).	Clinically diagnosed bulimia Nervosa	Participants were asked to write two letters to their bulimia, one addressing it as a friend and the other as an enemy. Analysed using a coding scheme.	To investigate the attitude of people with BN to their illness.
**120.** Serpell, L., Treasure, J., Teasdale, J. and Sullivan, V. 1999. Anorexia nervosa: friend or foe? *The International journal of eating disorders.* **25**(2), pp.177-186.	UK	34 letters collected from 18 patients (median age of 24.1 years)	Clinically diagnosed Anorexia Nervosa	Participants were asked to write two letters to their anorexia, one addressing it as a friend and the other as an enemy. Analysed using a coding scheme developed using grounded theory methodology.	To examine anorexics' attitudes towards anorexia nervosa
**121.** Shaw, L.K. and Homewood, J. 2015. The Effect of Eating Disorder Memoirs in Individuals with Self-Identified Eating Pathologies. *Journal of Nervous and Mental Disease.* **203**(8), pp.591-595.	Australia	24 women aged between 16 and 47 years	Self-reported ED.	Qualitative questionnaire and thematic analysis.	To explore the impact of eating disorder memoirs on individuals who have previously suffered or are currently suffering from an eating disorder.
**122.** Shillito, J.A., Lea, J., Tierney, S., Cleator, J., Tai, S. and Wilding, J. 2018. Why I eat at night: A qualitative exploration of the development, maintenance and consequences of Night Eating Syndrome. *Appetite.* **125**, pp.270-277.	UK	10 participants (3 male and 7 female). Age range = 20-71	Clinically diagnosed night eating syndrome (category of binge eating)	Semi-structured interviews analysed using a constructivist approach to grounded theory.	To explore the relationship between NES and the experience of emotion from the perspective of patients accessing a weight management service.
**123.** Siegel, J.A., Mendoza, R.R., Tesselaar, J.M., DeJesus, J., Elbe, C.I., Caravelli, N.S., Troy, L., Fenton, M., Victoria, B., Herrera, J. and Blashill, A.J. 2024. "I felt so powerful to have this love in me": A grounded theory analysis of the experiences of people living with and recovering from eating disorders while in diverse romantic relationships. *Body image.* **49**, p101709.	USA	66 people (45 cisgender women, 11 cisgender men, 9 nonbinary people and 1 transgender man) mean age = 27.46, SD = 5.97.	Self-reported ED.	Semi-structured interviews and grounded theory analysis.	To explore the ways that people living with and recovering from ED's experience their romantic relationships, with the specific objective to developing a novel theoretical framework, grounded in the experiences of people in diverse romantic relations to guide further research on the topic.
**124.** Simone, M., Askew, A.J., Beccia, A.L., Cusack, C.E. and Pisetsky, E.M. 2024. 'Let the ladies know': queer women's perceptions of how gender and sexual orientation shape their eating and weight concerns. *Culture, health & sexuality.* **26**(1), pp.108-125.	USA	105 queer women, age =23.6+/-3.4 years. 98 identified as cisgender and 7 as trans women/transfeminine	ED symptomology according to EDE-Q	Online study including open ended questions and reflexive thematic analysis	To explore lived experiences of queer women affected by eating and weight-related concerns.

**125.** Skårderud, F. 2007. Eating one's words, part I: 'Concretised metaphors' and reflective function in anorexia nervosa--an interview study. *European Eating Disorder Review.* **15**(3), pp.163-174.	Norway	10 female patients (age 16–35 years).	Anorexia nervosa according to DSM-IV criteria. 7 of the 10 participants had suffered from the restrictive subtype of anorexia, where the main symptom is restriction of food. The remaining 3 corresponded to the bulimic subtype (ANB), with episodes of bingeing behaviour.	Semi structured interviews and transcripts from therapy sessions. Qualitative text analysis.	To further understand the specific psychopathology of anorexia nervosa. A more precise aim is to develop a more precise language about the body's symbolic role in anorexia nervosa.
**126.** Skårderud, F. 2007. Shame and pride in anorexia nervosa: a qualitative descriptive study. **15**(2), pp.81-97.	Norway.	13 female participants aged 16-39 years.	Anorexia Nervosa according to DSM-IV. Eight of the 13 patients had suffered from the restrictive subtype of anorexia, the main symptom being restriction of food. The remaining five corresponded to the bulimic subtype (ANB), with episodes of bingeing behaviour.	Semi structured interviews and qualitative text analysis.	To define shame and describe types and subtypes of shame and their relations to symptoms and meaning in anorexia nervosa. The study will also describe the possible role of pride, as a contrasting emotional and cognitive experience
**127.** Smalley, V., Dallos, R. and McKenzie, R. 2017. Young women's experience of anorexia, family dynamics and triangulation. *Contemporary Family Therapy: An International Journal.* **39**(1), pp.31-42.	UK	6 female participants aged between 16 and 18.	Clinically diagnosed anorexia nervosa	Semi-structured individual interviews, a family sculpt and use of an adapted version of the adolescent separation anxiety test. Interpretative phenomenological analysis.	To explore the experience of triadic family relationships of six young women with a diagnosis of anorexia nervosa alongside a consideration of their attachment strategies.
**128.** Staite, E., Zaremba, N., Ismail, K., Macdonald, P., Treasure, J., Allan, J. and Stadler, M. 2018. 'Diabulima' through the lens of social media: a qualitative review and analysis of online blogs by people with Type 1 diabetes mellitus and eating disorders. *Diabetic Medicine.* **35**(10), pp.1329-1336.	UK	11 blogs (304 posts). No demographic information regarding bloggers available.	Self-reported ED or diabulimia.	Online blogs published between 2012 and 2017 authored by people who report having T1 diabetes and an ED or diabulimia. Thematic analysis.	To conduct a qualitative review of online blogs authored by people self-identifying as having type 1 diabetes and an eating disorder or 'diabulimia'. A term used by people with T1 diabetes to describe an eating disorder that is characterised by deliberate restriction of insulin to control weight.
**129.** Stewart, S.H., Brown, C.G., Devoulyte, K., Theakston, J. and Larsen, S.E. 2006. Why Do Women with Alcohol Problems Binge Eat?: Exploring Connections between Binge Eating and Heavy Drinking in Women Receiving Treatment for Alcohol Problems. *Journal of Health Psychology*. **11**(3), pp.409-425.	Canada	58 adult women age range 19-64. 18 participants completed interviews. 3 of the 18 participants also took part in a focus group.	Self-reported binge eating	Mixed methods – Questionnaire with a sub sample of participants completing qualitative interviews and a focus group. Analysis method unclear.	Explore the possibility that binge eating and heavy drinking may be highly co-prevalent because they reflect common mechanisms involving emotional regulation.

**130.** Stirling, A.E., Cruz, L.C. and Kerr, G.A. 2012. Influence of retirement on body satisfaction and weight control behaviors: Perceptions of elite rhythmic gymnasts. *Journal of Applied Sport Psychology*. **24**(2), pp.129-143.	Canada	8 retired female rhythmic gymnasts, age range 18-24.	Self-reported weight control behaviours: food restriction, calorie counting, use of laxatives and diet pill and excessive exercise.	Semi-structured interviews. Unclear analysis method.	To explore rhythmic gymnasts' perceptions of the influence of their retirement transition on body satisfaction and weight control behaviours.
**131.** Stockford, C., Stenfert Kroese, B., Beesley, A. and Leung, N. 2018. Severe and Enduring Anorexia Nervosa: The personal meaning of symptoms and treatment. *Women's Studies International Forum*. **68**, pp.129-138.	England	6 women, age range 33 - 48.	Clinically diagnosed anorexia nervosa. Symptoms of restriction and purging.	Semi-structured interviews and interpretative phenomenological analysis.	To explore the experiences of women with severe and enduring anorexia nervosa.
**132.** Tan, J, Hope, T. and Stewart, A. 2003. Anorexia nervosa and personal identity: The accounts of patients and their parents. *International journal of law and psychiatry*. **26**(5), pp.533-548.	UK	10 young women aged between 13-22 and 7 mothers and 1 mother/father.	Clinically diagnosed anorexia nervosa.	Pilot qualitative study using interviews. Unclear method of analysis.	To explore the different factors relevant to competence to make treatment decisions in anorexia nervosa, through the mechanism of allowing patients and their families to give accounts of their own experiences and difficulties with this, as opposed to imposing pre-existing criteria in attempts to test competence.
**133.** Thomsen, S.R., McCoy, J.K. and Williams, M. 2001. Internalizing the impossible: anorexic outpatients' experiences with women's beauty and fashion magazines. *Eating disorders*. **9**(1), pp.49-64.	USA	28 females aged between 18-43.	Clinically diagnosed Anorexia Nervosa. Anorexic/DE behaviours	In depth qualitative semi-structured interviews analysed using grounded theory approach.	To explore the ways in which anorexic women use and experience women's beauty and fashion magazines and to understand how that use influences their eating disordered thinking and behaviour.
**134.** Tierney, S. and Fox, J.R.E. 2010. Living with the anorexic voice: a thematic analysis. *Psychology and psychotherapy*. **83**(Pt 3), pp.243-254.	UK	21 female participants mean age of 22.1 years, SD=6.1.	Anorexia nervosa.	Individuals were invited to write about their life with an anorexic voice in the form or a poem, reflection, letter or descriptive narrative. Thematic analysis.	To investigate experiences of and reflections on living with an anorexic voice.
**135.** Warin, M.J. 2006. Reconfiguring relatedness in anorexia. *Anthropology & Medicine*. **13**(1), pp.41-54.	Australia, Canada and Scotland	Participants came from a wide variety of backgrounds and locations. No demographic details available.	Anorexia nervosa.	Multisite fieldwork, participant observations and interviews. Unclear method of analysis.	Examined the ways in which a group of people with a diagnosis of anorexia understood and experienced relatedness in their everyday lives.

**136.** Wasson, D.H. 2003. A qualitative investigation of the relapse experiences of women with bulimia nervosa. *Eating disorders.* **11**(2), pp.73-88.	USA	26 women age range between 20-59 years.	History of clinically diagnosed bulimia nervosa	Focus groups and individual interviews underpinned and analysed using grounded theory. Constant comparative method of analytic induction.	To describe the relapse experiences of women with BN through a qualitative analysis of their experiential accounts.
**137.** Weaver, K., Wuest, J. and Ciliska, D. 2005. Understanding women's journey of recovering from anorexia nervosa. *Qualitative Health Research.* **15**(2), pp.188-206.	Canada	12 women. Age range 14-63.	Anorexia nervosa and self-identified as recovered or recovering.	Feminist grounded theory. Individual interviews and comparative analysis.	Understand more about the subjective process of women's recovery in the context of family, community, or society.
**138.** Westwood, H., Lawrence, V., Fleming, C. and Tchanturia, K. 2016. Exploration of Friendship Experiences, before and after Illness Onset in Females with Anorexia Nervosa: A Qualitative Study. *PloS one.* **11**(9), pe0163528.	UK	10 female participants aged between 18 and 42.	Clinically diagnosed anorexia nervosa	Semi-structured interviews and thematic analysis.	To qualitatively explore the friendship experiences of individuals hospitalised for AN, both before the onset of their disorder and during the time they have been unwell.
**140.** Williams, K., King, J. and Fox, J.R. 2016. Sense of self and anorexia nervosa: A grounded theory. *Psychology and Psychotherapy.* **89**(2), pp.211-228.	England and Wales.	11 women aged between 18 and 60. Mean age = 28.	Lifetime history of anorexia nervosa as per DSM-V criteria.	A constructivist grounded theory methodology. Semi-structured interviews.	The aim of this study was to explore the nature of the relationship between the self and the eating disorder in individuals with a lifetime history of anorexia nervosa.
**139.** Wicksteed, A. 2000. Manifestations of chaos and control in the life experiences of individuals with eating disorders: explorations through qualitative email discourse. *Feminism and Psychology.* **10**(4), pp.475-480.	USA, Canada, UK and Germany	32 individuals age range between 14-56. Gender unclear.	Eating disorders	Qualitative email discourse	Unclear
**141.** Williams, S. and Reid, M. 2007. A grounded theory approach to the phenomenon of pro-anorexia. *Addiction Research & Theory.* **15**(2), pp.141-152.	Unclear.	In total, 176 online identities participated. Further demographic information not given to protect anonymity.	Anorexia.	Virtual ethnographic approach to observe communication in three pro-anorexic web forums. Grounded theory.	To use pro-anorexia websites and the language used within their message boards and online diaries as a source of information to look at the attitudes and feelings that the people involved have towards their anorexia, to investigate the phenomena of pro-anorexia sites and to find out why people believe in pro-anorexia

**142.** Williams, S. and Reid, M. 2010. Understanding the experience of ambivalence in anorexia nervosa: the maintainer's perspective. *Psychology & health.* **25**(5), pp.551-567.	USA, Canada, Spain, South Africa, Australia, New Zealand, Romania and India	13 females and 1 male aged 18-36.	Self-reported anorexic behaviours.	Online focus group to collect discussion data and email interviews. Interpretative phenomenological analysis.	The study investigated the experiences and understandings of those who wish to maintain their anorexia and looked at how these understandings may affect their treatment experiences.
**143.** Williams, S., Reid, M. and health. 2012. 'It's like there are two people in my head': A phenomenological exploration of anorexia nervosa and its relationship to the self. *Psychology & Health.* **27**(7), pp.798-815.	UK	14 participants. 2 males and 12 females. Age range between 21-50 years.	Self-identified AN or EDNOS. AN behaviour including strict dieting, episodes of bingeing or purging, laxative, diuretic or diet pill use or use of heavy exercise.	Online focus groups or E interviews. Interpretative phenomenological analysis.	To explore the lived experience of anorexia nervosa from the perspective of those who use pro-recovery websites for ED's.
**144.** Young, S., Paul, R., Stephen, T. and and Hay, P. 2015. The role of exercise across the lifespan in patients with anorexia nervosa: a narrative inquiry. *Advances in Eating Disorders.* **3**(3), pp.237-250.	Australia, USA, Switzerland and New Zealand.	24 female participants. Participants' age ranged between 17.07–69.92years, with an average of 31.45 years.	10 participants currently in treatment for AN, 7 partially recovered and 7 fully recovered, according to stringent criteria.	Semi structured interviews and narrative enquiry methodology.	To investigate the key features of exercise prior to the onset of AN and throughout the illness. It also aims to determine whether individuals are able to re-establish healthy exercise within recovery, and to provide insight on this experience
