# Supplementary material for: How Do the Psychological Functions of Eating Disorder Behaviours Compare with Self-Harm? A Systematic Qualitative Evidence Synthesis
Source: Healthcare (Basel). 2025 Aug 5;13(15):1914. doi: 10.3390/healthcare13151914 (PMC12346682; doi:10.3390/healthcare13151914)
Supplement: Supplementary file 1 [file healthcare-13-01914-s001.zip › Supplementary Material - Search Strategy .pdf]

Supplementary Material S2:

1. **Search Strategy (Embase)**

2. Eating disorder/
3. Anorexia Nervosa/ or Anorexia/
4. Bulimia/
5. Avoidant restrictive food intake disorder/
6. Purging disorder/
7. Binge eating disorder/
8. (eat* adj3 disorder*).tw,kf.
9. Anorexi*.tw,kf.
10. Bulim*.tw,kf.
11. EDNOS.tw,kf.
12. (binge adj3 eat*).tw,kf.
13. (over adj3 (eat* or exercis*)).tw,kf.
14. (compuls* adj3 (eat* or exercis*)).tw,kf.
15. (restrict* adj3 (eat* or food or calori*)).tw,kf.
16. Purg* .tw,kf.
17. (Misus* adj3 laxative*).tw,kf.
18. ((self-induc* or selfinduc*) adj3 vomit*).tw,kf.
19. (Compensat* adj3 behavio?r*).tw,kf.
20. or/ 1-18
21. Function*.ti.
22. Motivation/
23. Motiv*.ti.
24. Intent*.ti.
25. Incentive/
26. Incentiv*.ti.
27. Reason*.ti.

28. Drive/
29. Driv*.ti.
30. Caus*.ti.
31. Purpose.ti.
32. Explanation*.ti.
33. Why.ti.
34. Meaning*.ti.
35. Perception/
36. Perception*.ti.
37. Experience*.ti.
38. Account*.ti.
39. or/ 20-37
40. Qualitative research/ OR Qualitative analysis
41. Qualitative*.tw,kf.
42. Interview/
43. Interview*.tw,kf.
44. (("semi-structured" or semistructured or unstructured or informal or "in-depth" or indepth or "face-to-face" or structured or guide*) adj3 (discussion* or questionnaire*)).tw,kf
45. Focus group*.tw,kf.
46. Participatory research/
47. Participatory research.tw,kf.
48. Grounded theory/
49. (grounded adj1 (stud* or analys?s or theor*)).tw,kf.
50. (narrative* adj1 (analys?s or approach* or synthes?s)).tw,kf.

51. Conversation analys?s.tw,kf.
52. ((personal or self report* adj2 (account*)).tw,kf.
53. (phenomenolog* adj1 (stud* or analys?s or approach*)).tw,kf.
54. Phenomenology/
55. ethnograph*.tw,kf.
56. Ethnography/
57. ((life or lived or personal) adj1 experience*).tw,kf.
58. (lifeworld or life world or life stor*).tw,kf.
59. Personal experience/
60. (photovoice or photo voice).tw,kf.
61. Theme*.tw,kf.
62. or/ 39-60
63. 19 and 39 and 61
